# Staff experiences of a reablement approach to care for older people in a regional Australian community: A qualitative study

**DOI:** 10.1111/hsc.13331

**Published:** 2021-03-08

**Authors:** Hazel Maxwell, Marguerite Bramble, Sarah J. Prior, Anne Heath, Nicole S. Reeves, Annette Marlow, Steve Campbell, Douglass J. Doherty

**Affiliations:** ^1^ School of Health Sciences University of Tasmania Sydney NSW Australia; ^2^ School of Nursing Midwifery Charles Sturt University Bathurst NSW Australia; ^3^ School of Medicine University of Tasmania Burnie Tas Australia; ^4^ University College University of Tasmania Burnie Tas Australia; ^5^ School of Nursing University of Tasmania Newnham Tas Australia; ^6^ Family Based Care Tasmania Burnie Tas Australia

**Keywords:** care coordinator, direct care worker, experience, health service research, independence, reablement, regional, social care

## Abstract

Reablement is described as a person‐centred, goal‐directed intervention with a view to regain, maintain or improve the independence of older clients. Although evidence to support the use of reablement as a multidisciplinary, home‐based intervention for community‐dwelling older adults is increasing, there is limited knowledge about what it means for care staff who provide client‐based services. This study, which was nested in a larger program evaluation, used a descriptive qualitative approach to explore direct care staff and care coordinator experiences of translating a reablement training program into practice for older people in a regional Australian community. Two months after the training program four focus groups were conducted with 13 care coordinators to assimilate staff experiences with development of care plans, systems, processes and practices of reablement. In addition, four direct care staff took part in individual interviews, which centred on eliciting their experience using the reablement approach with clients. Results from the care coordinator focus groups and the direct care staff interviews highlight the importance of reablement staff training and the involvement of staff in the development and delivery of a reablement approach to client‐centred care. A number of organisational and client‐centred challenges such as communication, functional partnerships, staff education and resourcing are also uncovered in this research into the development of a reablement‐focused care service in a regional setting. Overall there is support for the dominating discourse around healthy ageing and the policy approach of ageing in place to support wellness.

**What is known about the topic?**

- Reablement is a person‐centred, goal‐directed intervention used to regain, maintain or improve the independence of older people
- Community‐dwelling older adults value reablement
- Reablement fits with the dominating discourse around active ageing and the policy approach of health and social investment in wellness

**What the paper adds?**

- Adds to the limited knowledge about what reablement means for care staff who provide client‐based services
- Provides knowledge about innovative approaches to reablement training for staff
- Provides an insight into a regional approach to reablement delivery within a single community‐based organisation using a qualitative research approach

## 1 INTRODUCTION

With a growing population of older people world‐wide, new health and social care services are required which can promote and include active client involvement (Bloom, 2019; Smeets et al., 2020). Reablement is a relatively recent healthcare approach that has an emphasis on intensive, goal‐oriented and interdisciplinary rehabilitation for older adults who are at risk of functional decline (Cochrane et al. 2013; Doh et al. 2020; Liaaen & Vik, 2019; Smeets et al., 2020; Wilde & Glendinning, 2012). There has been some concern that reablement programs are only slight variations of rehabilitation programs based on the definitions of rehabilitation by the World Health Organisation (2011) and the Department of Health in the UK (Legg et al. 2016). However, the key difference, as described by Hjelle et al. (2016), is the major focus for reablement on home‐based rather than institutionalised care (Hjelle et al. 2016). Evidence also suggests that older people prefer to age in place, that is, remain in their homes for as long as possible with the provision of services to enable independence (Boldy et al. 2010; Smeets et al., 2020; Wiles et al. 2012). Encouraging this more active approach to ageing in place is important from both a health service and client perspective.

As a person‐centred, empowerment‐based (Smeets et al., 2020) approach to care, the reablement approach relies on the development of a qualified workforce that can meet the increasing complexity of and demand for aged care services. In Australia, reablement also fits with the philosophy of Consumer Directed Care (CDC) with all community home‐care packages now funded on a CDC basis (Australian Government Department of Health Ageing & Aged Care, 2019; Cash et al. 2017). CDC is an Australian healthcare model in which the client is given full independence in relation to decisions around their care.

A 2019 review of Australian health services concluded that while Australian healthcare systems perform well by international standards, they continue to fall short of providing equitable access to care for all Australians through primary health and community care services (Calder et al. 2019). In community care services, the implementation of the reablement approach is timely in that it challenges the traditional care and support worker role of “maintenance” and “support,” shifting to a proactive and restorative approach to care, thus providing clients with the opportunity to age in place, be active and participate more socially and societally (Australian Association of Gerontology, 2020; Campbell et al., 2019; Prior et al., 2020). The workforce implications of supporting wellness and reablement must also be addressed so that changes in values, attitudes and organisational culture are embraced. All direct care and support staff can then fully implement reablement in practice based on the CDC principles of service user self‐determination, empowerment and choice (Laragy & Allen, 2015).

While there is broad agreement that program design and funding model changes to CDC should include a wellness and reablement focus, the issue of inequitable access in rural and regional areas for people living with chronic illness and disability drives the need for reform (Lawn et al. 2017). This includes increased investment in staff training to address the diverse needs of clients (Lawn et al. 2017). From an economic and business perspective, measuring the impact of aged care programs is also a key concern, particularly under individualised funding models where service providers compete to attract “consumers” by promising quality care (Cardona, 2018). Added to these challenges are the difficulties of training direct care and support staff in providing effective, team‐based support for clients who are geographically dispersed and socially disparate (Low et al., 2018; Prior et al., 2020).

Previous qualitative studies focused on reablement have suggested that clients see reablement as a positive approach (Wilde & Glendinning, 2012) that makes a significant difference to their lives (Godfrey et al. 2005) through greater improvement in their self‐care, home management and mobility (Tinetti et al. 2002) as well as improved participation in society (Wilde & Glendinning, 2012). Reablement in a client's home enables independence and autonomy and the support clients receive from reablement increases their confidence to safely perform and participate in everyday and social activities (Hjelle et al. 2016).

A core factor in a reablement approach is ensuring community‐based care organisations, which include direct care staff and care coordinators as well as organisational leads and health professionals, can collaborate with each other and with clients, to determine and work towards the best outcomes for clients. Termed interdisciplinary collaboration (Birkeland et al. 2017; Hjelle et al., 2018; Moe et al., 2019), this form of collaboration focuses on the relationships and mutual trust between various disciplines (Steihaug et al. 2014). It involves using and sharing one's own capabilities to plan, communicate, share responsibilities and decisions (Thylefors et al. 2005; White et al. 2013) and to support clients in their restorative process. This process consists of encouraging clients to define their own goals and focus on participation in their own reablement process rather than having these decided by a third party (Hjelle et al., 2018; Liaaen & Vik, 2019; Moe et al., 2019; Rostgaard, 2018; Smeets et al., 2020).

Providing care within a reablement focused system can have a positive impact on employees by increasing job satisfaction and reducing staff turnover rates compared to those working within traditional models of care (King et al. 2012; Rostgaard et al., 2016). The role of coordination staff in community‐based care and support organisations can vary between working directly with clients to developing client goals and planning ongoing support and working directly with care workers to facilitate the needs of the clients and their families. All employees work together to provide services that are responsive, person centred and respectful. These services include support with home maintenance, domestic assistance and the provision of personal care, respite care and social support. A previous program aimed at improving the care of chronically ill patients in the community‐defined care coordination as “the deliberate organisation of patient care activities between two or more participants involved in a patient's care to facilitate the appropriate delivery of health care services” (McDonald et al. 2007, p. 1). This aligns with the notion that care coordinators and direct care workers collaborate, sharing knowledge and ideas, to provide best practice reablement‐focused care for their clients while providing a working environment conducive to staff needs.

## 2 STUDY AIM

The aim of this study, which was nested in a larger evaluation of a teaching program for staff (Prior et al., 2020), is to explore direct care staff and care coordinator experiences of translating a reablement‐focused program into practice for older people in a regional community in Australia. The study comprised community‐based organisation staff participating in training activities related to incorporating reablement within their scope of practice and reflecting on this afterwards. The training was health department funded.

### 2.1 Study intervention

Two preliminary workshops were held with a group of eight direct care staff members, who worked with the research team to develop themes around how reablement in practice could be taught and evaluated. This group of staff from the community organisation volunteered their involvement in working with the research team to develop materials for the training sessions based on their own experiences and organisational strategy. These materials included videos of their own stories about reablement and PowerPoint presentations addressing the main themes around reablement within their community. During the workshops, the participants reviewed the activities of daily living (ADLs) that were their focus of care, and their relationship with reablement. Examples of these ADLs included walking, eating and drinking, sleeping and resting and selecting clothes. Participants were then encouraged to give examples of reablement in action and as a result of these discussions between researchers and staff, each of the participants recorded at least one reablement in practice example as a video. These videos were then included as a learning and teaching resource within the reablement training program.

The reablement training program was subsequently conducted for a further 166 staff members at the organisation. Staff attended two, 2‐hr reablement training sessions (approximately 2 months apart, starting in March 2018) at the premises of the organisation. The two training sessions were designed by the research team and approved by the leadership team of the community‐based organisation prior to commencement. Each training session contained two video examples of reablement in practice from their colleagues within the organisation. These videos prompted in‐depth discussion around reablement by the participants. Each training session was conducted by two members of the research team. The materials used in the training were based on the identified ADLs, with examples developed and used in consultation with staff at the organisation to ensure the case studies used were appropriate, relevant and relatable.

## 3 METHODS

Ethical approvals were obtained from the Research Ethics Committee of the Regional Australian University (Reference H0017264 and H0017139). Qualitative research methods were adopted involving focus groups and in‐depth individual interviews with a convenience sample of staff who provided written informed consent. Two months following the training program, four focus groups were conducted with 13 care coordinators to assimilate direct care staff experiences with development of care plans, systems, processes and practices. These focus groups (approximately an hour in duration) held fortnightly across an 8‐week period, centred on how the organisation was supporting the introduction and development of a reablement culture within all levels of care across the workplace. Each focus group was facilitated by two members of the research team who had no direct involvement with the organisation and no direct involvement with local community care. Adequate focus group sample size generally comprises 6–12 participants, however, as care coordinators within the organisation typically work with a variety of different types of direct care workers across different localities, we aimed to include a representative sample to ensure all views were considered.

In addition to a convenience sample, four direct care staff took part in individual interviews, which centred on eliciting their experience using the reablement approach with clients. These were held approximately 2 months post training. These four staff interviews (25 min to 1 hr in duration) were conducted in a place of each participant's choosing by one member of the research team. The interviews and focus groups were recorded via an audio recorder and thematic analysis was performed using Nvivo software. The interview questions were designed to explore attitudes and knowledge of reablement and its delivery through the organisation.

Thematic analysis of the data was conducted according to the method defined by Attride‐Stirling (2001). This approach involves the development of basic themes which are further refined further into organising themes with the global themes deduced from these data. Global themes bring together the basic and organising themes into one or two overarching categories. A system of coding was developed, thereby allowing data to be divided into segments and allocated accordingly. Refining and arranging these segments into basic themes gave a preliminary structure for our overall themes. Interpretation of these five global themes (see Table 1) developed from this research makes up our discussion.

**TABLE 1:** Global themes and working definitions

Theme	Definition
Understanding through education	The provision of education around reablement enables staff to align their practice with this focus. Understanding reablement from an organisational perspective helps staff to further develop their own skills and confidence to deliver reablement‐specific activities in their role.
Valuing client‐centred care	Ensuring clients (and their families/support networks) are at the centre of all decisions that are made, including initial planning, follow up and ongoing support are something staff value highly.
Reablement is rewarding	Working within a reablement framework provides a sense of achievement for care coordinators through helping clients work towards independence.
Conversation enables action	Conversation is needed to provide appropriate and reablement‐focused care and support to clients and their families and to ensure that staff are adequately supported to undertake their roles.
Partnerships in care	Care coordinators consider their relationships with stakeholders, including direct care workers and clients, as partnerships.

## 4 FINDINGS

The five themes evident in the focus groups and individual interviews included understanding through education and training, valuing client‐centred care, reablement is a rewarding approach, conversation enables action and partnerships in care. Within these topics some important positive and negative impacts of introducing reablement into a community‐based organisation via a training program emerged. After the education sessions, direct care staff could explain their understanding of reablement.

It’s the only philosophy for care, I believe, because, you know, not just for …, but for everywhere. And it really is about giving that to people what is rightly theirs, and it’s about empowering them, and it’s about strengthening and building relationships. (INT P1)

Following the reablement training, staff felt supported within their organisation to develop a reablement view of supporting older clients that was important socially, physically, mentally and emotionally, to maintain independence in clients and to create a sense of “staying useful” for staff. A clear thought process around the reablement concept is articulated below:

Since the teaching… I have been more mindful of encouraging independence and I am very much a doer and I’m very much, look, I can just do that for you… So, I had to be really aware of stepping back, and if it takes my client ten minutes to do something and it takes me half a minute to do something, I really do consciously have to step back and allow that ten minutes, rather than get it done in half a minute yourself and it’s done. (INT P2)

The strongest theme evident throughout the focus groups and individual interviews was the importance staff placed on valuing client‐centred care. This reflects the discussions across all four focus groups about keeping clients at the centre of decision making and planning. According to some focus group members, valuing client‐centred care is not a skill that an individual can learn but a fundamental belief. A participant explained “You can teach a skill, but you can't teach a value” (FG2 P8). It is providing care that is respectful and responsive to individual client needs and is ingrained, not necessarily something that can be learnt. It's about “Helping someone to be the best that they can be” (FG1 P3).

Direct care staff explained that clients receiving reablement appeared more valued if they worked together with workers in a partnership rather than a traditional worker/client relationship. Yet, there was some concern expressed by direct care staff about this approach particularly if the staff themselves became unnecessary if, and when, clients were able to do tasks they previously needed help with themselves.

A number of participants felt that ensuring communication channels remained open across the organisation was important in building relationships (between staff and clients and within the organisation) and this was a key factor in the success of reablement for individual clients and their families. Staff spoke about the role of conversation in reablement and how care coordinators could better support their direct care staff by giving them opportunities to have conversations with them, in person, to reflect on their experiences, ask for advice and identify any issues on a regular basis. One participant stated that they needed to, “catch up with our team and have the small meetings and we chair that meeting” (FG3 P6).

Care coordinators identified a number of important barriers to these peer support conversations which included access and time. The physical location of the offices of care coordinators was suggested to be a main barrier for direct care staff as they are located in the city centre where paid parking is required, facilities are inappropriate and an overall sense of intimidation was perceived. Whether or not these conversations should be a part of the working hours for both care coordinators and direct care staff was also discussed as paid time is often limited to direct client‐focused activities. Sharing successful reablement stories as part of evaluation and reflection was thought to be an important part of creating a reablement culture within the organisation. One participant questioned, “How do we know reablement is happening, if we don't have these conversations?” (FG2 P7)

The findings also show that staff have their client's best interests in mind with discussions reflective of the importance care coordinators place on their relationships with their clients, which at times appeared to be at the detriment of relationships with direct care workers. A care coordinator proposed that “your team is your client and family, not other professionals”. (FG4 P5)

Discussions around duty of care, respect, value and positively impacting people's lives really highlight the compassionate nature and genuine effort in helping to regain, or gain, some independence for clients through utilising reablement. A direct care staff member stated, “It's also a form of reablement to make choices. I give the clients choices to choose from without them even really being aware that they've just had to make choices.” (INT P3) This quote illustrates how a reablement approach helps clients to regain independence.

Although not always directly involved in the care and support of clients, care coordinators felt they could empower clients by ensuring the care planning involved better understanding of what the clients want and need. This, in turn, would also support direct care workers to better partner with their clients, “walking the journey with the client”. (FG3 P1)

It was discussed by the research participants that some clients, families and direct care workers were passive in their approach to reablement and that the role of care coordinators was to form relationships with direct care workers that foster a safe and collaborative environment. The ability of the care and support workers after the training to translate, understand and implement reablement into practice across various areas of care is described in the narrative of the care or an ageing couple:

He’s really willing to do the gardening, that’s fantastic, but he just needs someone to mow the lawns. So, he sits on his wheelie‐walker, and he weeds all the garden, and he puts in his veggie plants and stuff. He can’t get about without walking with a walking frame or with his walking stick, but he can still drive the car, and she can still get the groceries in and out of the trolley and stuff like that, into the car. So, together they work as a team, they’re reenabling each other, and all they really needed was someone to come and to do some of those difficult things that they can’t do, with the home and garden. (INT P1)

However, the picture was not all positive and there were some concerns and issues with implementing reablement within the organisation, such as staff thinking “clients would think that their support workers wouldn't come (and provide support) if they were doing things themselves”. (INT P4) Another participant indicated issues around workload suggesting that her “biggest fear … is that it's so difficult already, you know. We're all time poor”. (INT P1) This participant further stated that “Reablement can be quite confronting for some people…depending on their circumstances…so, for some people it might be a different concept. They might see it as someone completing a task and not someone walking along their journey with them”. (INT P1)

A number of care coordinators described initially feeling “lost” and that the main barrier to a reablement culture was a lack of education, particularly around the words and language used and the organisational policies and processes. Developing methods for shared understanding between care coordinators and clients and their families for the development and interpretation of care plans was a solution put forward to improve experience. One person articulated that they needed to be “sharing successes and sharing challenges… connection with purpose”. (FG4 P3)

A lack of structured human resource systems and direct supervisory arrangements were also factors that care coordinators felt influenced their ability and the capability of their direct care staff to provide ongoing reablement‐focused support. One participant explained “We are on a road trip with no map”. (FG2 P10) The discussion centred on the lack of current human resources staff and formal human resources processes from both a care coordinator and direct care worker point of view.

## 5 DISCUSSION

These findings demonstrate that in order to adopt reablement in practice, all care staff and the organisation that supports them, need to adopt behaviours that are concordant with its principles. This requires “doing with, rather than doing for” the client. Next steps involved identifying behaviours that needed to be changed at the functional level for all clients. The reablement approach adopted was via ADLs and successfully involved supporting clients in walking, breathing, elimination, eating and drinking, movement, sleep and rest activities, selecting clothes, learning and discovery of new activities, body temperature regulation, hygiene activities, avoiding dangers, communicate tasks, worship, work accomplishment and leisure and play. Reablement principles were applied and embedded in the creation of the learning and teaching resources and the teaching program as well as the evaluation of the impact of reablement across the organisation. Most importantly the research team worked with the organisation to develop the learning and teaching resources, mirroring appropriate reablement practices of working with clients to achieve goals.

Following the training program, staff mostly discussed reablement in glowing terms and appreciated the support their organisation was providing for training and implementation. This improved motivation and gave a much needed “morale boost”, an important benefit of reablement which has been noted in other international studies (Rostgaard, 2018). Recent research from Denmark shows that care and support workers working intensively with reablement (daily or at least once daily) experience a number of benefits (Rostgaard, 2018). The care workers were more likely to find giving care rewarding, they found that they received support from their managers, they found that older people's needs were better met and they were less likely to want to leave their jobs (Rostgaard, 2018).

The staff experience of the reablement training provided a unique insight into both organisational challenges as well as client‐centred challenges in the development of a reablement‐focused care service. The findings from this study also indicate a need for an understanding among care coordinators, direct care workers and the client's family in order to work effectively and in the best interests of the client. It is important therefore that there is communication around what a client can achieve independently, ensuring that staff do not take control over activities that a client can do for themselves. Instead staff need to use this information to complement tasks that clients are unable to perform themselves. This aligns with previous international research (Hjelle et al., 2018; Rostgaard, 2018; Smeets et al., 2020) that discussed the importance of collaboration and working together to achieve the best outcomes for clients. According to our results, ensuring communication channels remain open and clear and clients feel able to develop and build relationships is a factor in the success of a reablement framework of care for individual clients and their families.

Data demonstrate the importance of the relationship between care coordinators and direct care workers and how in future this could be improved to further enhance and deliver client care and reablement education within the organisation. Suggestions for improvement from the staff experience included opportunities for regular meetings, seeking advice and discussing workforce‐related and client‐related issues. This is consistent with previous research (Rabiee & Glendinning, 2011) that demonstrates the features, content and delivery of reablement services (such as a limited potential for staff to be independent, a lack of continuity of support and time pressures) may detract from its effectiveness in an organisation.

Discussions and reflections give professionals the opportunity to learn from each other and develop a shared professional platform that includes a rehabilitative approach, interdisciplinary competence and relationships (Hjelle et al., 2018; Jokstad et al., 2019; Low et al., 2018). In the focus groups and interviews with care coordinators, direct care workers alluded to feeling somewhat isolated in the community and missing that link to their peers. Having daily team meetings, a chance to work more closely together and to learn from one another in some capacity may provide cohesion that is currently lacking for some direct care workers and can help to enhance the relationships between direct care workers and care coordinators. Rabiee and Glendinning (2011) found that having regular team meetings, peer support and supervision sessions reinforced reablement delivery and kept staff informed and motivated. Other recent research indicates that one of the most appreciated elements of reablement involves activities, where staff can learn from each other by exchanging stories of successful experiences with one another often using narrative and role‐play (Low et al., 2018; Smeets et al., 2020).

Reablement education and training is valuable not only to teach the principles and value of reablement but also to reinforce the current independence‐based practice for staff and their clients (Smeets et al., 2020). Throughout the focus groups, care coordinators discussed the role of direct care workers working directly with clients. They indicated this role requires a good understanding and practice of reablement, with the skills to work closely with the client, providing support and motivation to allow them to find a new level of independence or regain their previous independence. The role of care coordinators should include the emphasis of the importance of continuous reablement training and provide staff with opportunities to continue learning and understanding reablement. Care coordinators also need to ensure ongoing team meetings and direct supervision are regularly provided, as necessary. This would reinforce the reablement approach and assist in curbing any resistance staff may have in delivering the new approach to clients (Rabiee & Glendinning, 2011). Norwegian and Danish studies have shown that the philosophy of reablement can co‐exist alongside pre‐existing philosophies of care and this does not need to be a barrier to reablement delivery (Moe et al., 2019).

Staff education and professional development programs in other settings such as nursing and aged care have been shown to have a positive impact on behaviour changes, with staff adopting a broader perspective and being more capable of identifying factors and effective strategies to support their patients (McPhail et al. 2009). By maintaining the highest standards of patient care through an ongoing commitment to staff education, best practice can be promoted (East & Jacoby, 2005). Standard practice takes time and as our results show, when staff are supported and provided with education and training opportunities, they are able to apply their individual learnings to change other people's views on reablement with the hope of reablement becoming best standard practice.

In addition, the findings of this study need to be contextualised within the Australian setting in which aged care is hampered by a disconnected workforce across many boundaries, perpetuating duplication and inefficiency (Lawn et al. 2017). To date, care and support workers have often been excluded from training, particularly in the area of supporting behavioural change. Also emerging is the requirement for new directions in creating effective systems of care and support that enhance the care and support worker's role. This would address the issue of services working in isolation, and clients often being over serviced but underserved (Lawn et al. 2017). Across Australia generally, provision of an integrated and coordinated workforce across systems to identify and respond to early deterioration in health status is not currently in place (Dickinsen & Carey, 2017; Lawn et al. 2017).

Enhancing the care and support worker's role in practice is timely given increasing demands on human and financial healthcare resources in community care (Hjelle et al. 2016; Lawn et al. 2017). In a qualitative study in the UK, the ideal reablement worker was described by managers as someone with a good understanding of the concept and practice of reablement, with the skills to stand back, observe and assess their clients’ potential for independence, and work closely with them to provide the support they needed to reach their potential (Rabiee & Glendinning, 2011).

Evidence available from other countries shows significant improvements in clients’ self‐perceived activity performance and satisfaction with performance, however, no significant improvements were noted in health‐related quality of life or physical capacity (Tuntland et al. 2015; Winkel et al. 2015). Economic evaluations of reablement programs showed, when compared to traditional care, cost savings of up to $12,500 AUD per person over a 5‐year period and approximately $3000 AUD per person over 2 years in separate studies (Lewin et al. 2013, 2014). A further economic evaluation by Kjerstad and Tuntland (2016) found a significant decrease in the need for long‐term home‐based care services following a time‐limited period within a reablement program, thus reducing the long‐term expenditure (Kjerstad & Tuntland, 2016).

The study is restricted to a single community‐based care organisation in a regional setting and did not include nursing or allied health professionals. As a qualitative study the ability to generalise findings is limited, however, similarities with findings from a range of international studies indicate that a relatively low number of participants in the sample has not detracted from some valuable insights into reablement development and training. This study is also limited to staff perspectives of reablement education and implementation and does not focus on client and family perspectives (Jakobsen et al., 2019). These different and equally valuable views require further future investigations in regional and urban contexts.

## 6 CONCLUSION

The current study shows that the direct care staff and care coordinators’ perspectives and experiences are vital in understanding and introducing a reablement‐based care system. With appropriate training and education in reablement, practice is shown to be meaningful and the concept of reablement can be clearly understood and articulated. Furthermore, it would appear that the dominating discourse around healthy ageing and the policy approach of “ageing in place” provide a strong case for supporting reablement and reablement training in community care. There is a need for greater commitment and investment from governments to develop a well‐trained, well‐paid and well‐supported workforce to ensure reablement can be delivered effectively and consistently especially in the light of challenges such as the increasing casualisation of the workforce and increasing demands on existing staff. Reablement is claimed to have the potential to increase the quality of life for older people and to make community care systems more sustainable by reducing the need, and thus the costs, for care. So far however, the evidence is limited, and more research is needed to confirm early positive findings.

## CONFLICT OF INTEREST

No conflict of interest exists.

## AUTHOR CONTRIBUTION

Study design: HM, MB, AM, AH, SC, DD, SP, NR. Data collection: NR, AM, AH, SC, SP. Data analysis: HM, NR, SP, MB, SC. Study supervision: HM, MB, SP, SC, AM, DD. Manuscript writing: HM, MB, AM, SC, SP, NR, AH, DD. Critical revisions for important intellectual content: HM, MB, AM, SC, SP, DD, NR, AH.

## Data Availability

The data that support the findings of this study are available on request from the corresponding author. The data are not publicly available due to privacy or ethical restrictions.
